# Identification of Combined Genetic Determinants of Liver Stiffness within the SREBP1c-PNPLA3 Pathway

**DOI:** 10.3390/ijms141021153

**Published:** 2013-10-22

**Authors:** Marcin Krawczyk, Frank Grünhage, Frank Lammert

**Affiliations:** Department of Medicine II, Saarland University Medical Center, Homburg 66421, Germany; E-Mails: marcin.krawczyk@uks.eu (M.K.); frank.gruenhage@uks.eu (F.G.)

**Keywords:** adiponutrin, gene variant, hepatic fibrosis, sterol regulatory binding protein 1c

## Abstract

The common *PNPLA3* (adiponutrin) variant, p.I148M, was identified as a genetic determinant of liver fibrosis. Since the expression of *PNPLA3* is induced by sterol regulatory element binding protein 1c (SREBP1c), we investigate two common *SREBP1c* variants (rs2297508 and rs11868035) for their association with liver stiffness. In 899 individuals (aged 17–83 years, 547 males) with chronic liver diseases, hepatic fibrosis was non-invasively phenotyped by transient elastography (TE). The *SREBP1c* single nucleotide polymorphisms (SNPs) were genotyped using PCR-based assays with 5′-nuclease and fluorescence detection. The *SREBP1c* rs11868035 variant affected liver fibrosis significantly (*p* = 0.029): median TE levels were 7.2, 6.6 and 6.0 kPa in carriers of (TT) (*n* = 421), (CT) (*n* = 384) and (CC) (*n* = 87) genotypes, respectively. Overall, the *SREBP1c* SNP was associated with low TE levels (5.0–8.0 kPa). Carriers of both *PNPLA3* and *SREBP1c* risk genotypes displayed significantly (*p* = 0.005) higher median liver stiffness, as compared to patients carrying none of these variants. The common *SREBP1c* variant may affect early stages of liver fibrosis. Our study supports a role of the SREBP1c-PNPLA3 pathway as a “disease module” that promotes hepatic fibrogenesis.

## 1. Introduction

Liver fibrosis is a trait driven by genetic and environmental determinants [1,2]. Since liver biopsy represents the “gold standard” method for quantifying liver fibrosis, the majority of previous studies aiming to define the genetic background of fibrosis were based on biopsy samples. Nevertheless, liver biopsy may be associated with complications [3] and costs, which renders recruiting large cohorts for well-powered genetic analyses troublesome [4]. Hence, in our latest candidate-gene study, we availed of transient elastography (TE) to phenotype a cohort of patients with chronic liver diseases [5]. This study demonstrated that carriers of the common adiponutrin (*PNPLA3*) variant, p.I148M, present with increased liver stiffness levels [5]. The *PNPLA3* polymorphism was previously identified in a genome-wide association study (GWAS), which showed that carriers of the risk allele are prone to hepatic fat accumulation and non-alcoholic fatty liver disease (NAFLD) [6]. The association was then replicated in additional NAFLD cohorts [7–12] and extended to alcoholic liver disease [13–15], as well as severe liver phenotypes resulting in hepatic fibrosis and cirrhosis [5,13,15–17]. Finally, cirrhotic carriers of the *PNPLA3* variant are at increased risk of hepatocellular carcinoma (HCC) [18–21], and in patients with HCC, this variant is associated with poor prognosis [22]. The above results, substantiated in the latest meta-analysis [23], establish the p.I148M adiponutrin variant as a common determinant of chronic liver injury leading to progressive fibrosis [24]. The recent study by Kumari *et al.* [25] demonstrated that *PNPLA3* p.I148M is a “gain-of-function” variant, which results in higher lysophosphatidic acid acyltransferase activity and increased hepatic diacylglycerol synthesis. Moreover, further genetic studies associated the variant with some metabolic traits, in particular related to lipid metabolism [26–29].

The above studies focused, however, on investigating primarily the *PNPLA3* locus. Indeed, the analysis of selected single polymorphisms within candidate genes can reveal variants associated with common traits; nevertheless, the investigation of genetic susceptibility within specific pathways regulating the expression of these genes might provide further insights into disease predisposition. As highlighted lately, such regulatory pathways could play a pivotal role in the development of organ-specific fibrosis [30]. Of note, Huang *et al.* [31] described a pathway of adiponutrin expression in mouse liver that is induced by carbohydrates. As shown in their study [31], the pathway includes the activation of nuclear receptors, namely, liver X receptor (LXR) and retinoid X receptor (RXR), which, in turn, increases hepatic expression of sterol regulatory element binding protein (SREBP1c). Upon ingestion of carbohydrates, SREBP1c induces the transcription of *PNPLA3* and increases the synthesis of fatty acids (FA) in the liver [31]. This observation in mice was substantiated by the analysis of human hepatocyte cell lines by Dubuquoy *et al.* [32], who showed that SREBP1c is the master regulator of hepatic *PNPLA3* expression in humans, as well. In short, SREBPs are transcription factors serving as central regulators of lipid and cholesterol metabolism [33–36]. The major SREBP isoforms, SREBP1a, SREBP1c and SREBP2, are encoded by two different genes [37]. Interestingly, previous studies demonstrated that two *SREBP1c* variants, rs2297508 and rs11868035, affect glucose and lipid metabolism in humans [38–41].

Hence, in the current study, we assess the influence of these common genetic variants within the *SREBP1c* gene on liver stiffness in a cohort of patients with chronic liver diseases.

## 2. Results

### 2.1. Phenotypic Characterization by Transient Elastography (TE)

**Figure 1:**
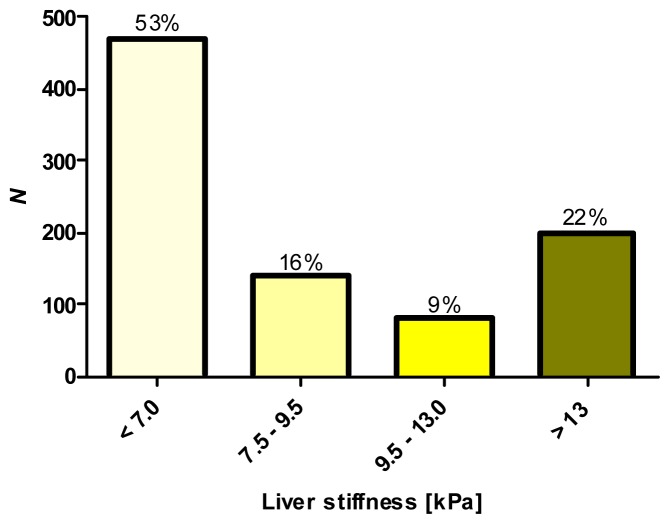
Distribution of transient elastography (TE) measurements in patients (*n =* 899) with chronic liver diseases. Most of the individuals (53%) included in the study presented with low liver stiffness, as defined by TE < 7 kPa, and 25% of patients had mild/advanced liver stiffness. The remaining 22% presented with TE levels ≥13 kPa, consistent with liver cirrhosis [42].

The median TE level in our cohort was 6.8 kPa (range 2.2–75.0 kPa). As presented in our previous publication [5], we observed a significant (*p <* 0.01) correlation (ρ = 0.74) between liver stiffness and histopathological fibrosis stages. TE levels also correlated with serum alanine aminotransferase (ALT) (*p* = 0.041) and aspartate aminotransferase (AST) (*p* = 0.001) activities. Figure 1 shows that most patients (>50%) presented with no or mildly increased liver stiffness values (*i.e*., TE values <7.0 kPa). On the other hand, TE values ≥13.0 kPa, indicating liver cirrhosis [42], were present in 22% of patients. The remaining 25% of the cohort presented with moderate liver fibrosis.

Among cirrhotic patients (*n =* 201, females *n* = 70, age range 19–82 years), 126 patients (62.6%) suffered from viral and 75 individuals (37.4%) from non-viral liver diseases (see Table 5 for details). Comparison of patients with chronic viral and non-viral liver diseases did not show significant differences in liver stiffness values (non-parametric *p* = 0.60).

### 2.2. The *SREBP1c* rs11868038 Variant Increases Liver Fibrosis

Overall, we achieved >98% genotyping success. The genotype frequencies of the *SREBP1c* polymorphism rs11868035 (*i.e*., (CC) = 87, (CT) = 384, (TT) = 421), rs2297508 (*i.e*., (GG) = 140, (GC) = 436, (CC) = 315) and the *PNPLA3* variant, p.I148M ((CC) = 481, (CG) = 348, (GG) = 63) did neither differ from the frequencies deposited in the *Entrez* database for Caucasian cohorts (*p* > 0.05) nor deviated from Hardy-Weinberg equilibrium (HWE) (all *p* > 0.05), indicating robust genotyping.

**Figure 2:**
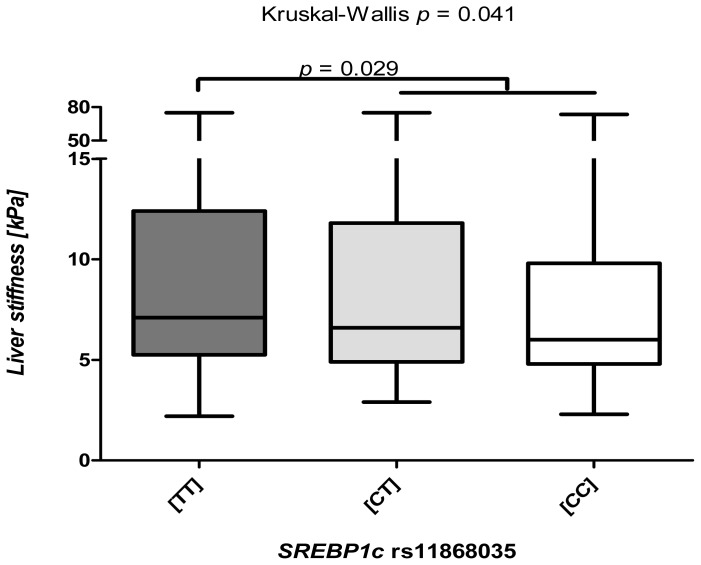
Box-and-whisker plots illustrating TE levels (liver stiffness (log kPa)) in carriers of different *SREBP1c* rs11868035 genotypes. Liver stiffness differs significantly (*p* = 0.041, non-parametric ANOVA) between carriers of *SREBP1c* genotypes. In particular, individuals carrying the genotype (TT) display significantly (*p* = 0.029) advanced liver fibrosis, as compared to carriers of genotypes (CT) and (CC).

As shown in Figure 2, median TE differed significantly (*p* = 0.041) between carriers of specific *SREBP1c* rs11868035 genotypes. The carriers of the (TT) genotype presented with significantly (*p* = 0.029) increased liver stiffness, as compared to individuals with (CT) and (CC) variants. On the other hand, the rs2297508 polymorphism was not associated with TE levels (all *p* > 0.05).

### 2.3. The *SREBP1c* Polymorphism Is Associated Predominantly with Low TE Levels

To further explore the effect of the *SREBP1c* polymorphism rs11868038 on hepatic stiffness, we performed a case-control analysis and compared the allele and genotype frequencies between individuals with absent or low fibrosis (*i.e*., TE < 7.0 kPa) and patients presenting with moderate and severe fibrosis (TE ≥ 7.0 kPa). Tables 1 (allele distribution) and 2 (genotype distribution) summarize the results of this analysis.

As shown in Table 1, the (T) allele was more prevalent among individuals with enhanced fibrosis as compared to patients with TE values <7.0 kPa (72% *vs.* 66%, *p* = 0.009). Conversely, the comparison of genotype frequencies (Table 2) revealed that the (TT) genotype was more frequent in the subgroup of patients with TE ≥ 7.0 kPa, as compared to the remaining cohort (51% *vs.* 43%, *p* = 0.009). On the other hand, we were not able to detect any association between the *SREBP1c* variant and liver, as staged by liver biopsy (*p* > 0.05).

### 2.4. The Common *SREBP1c* Variant Increases Liver Stiffness Independently of Other Profibrogenic Factors

To identify additional non-genetic risk factors for developing liver stiffness values ≥7 kPa, we performed logistic regression analyses. Tables 3 and 4 present the results of these analyses.

As shown in Table 3, the *SREBP1c* (TT) genotype and age, but not the adiponutrin variant, BMI, alcohol consumption or gender increased the risk of developing TE ≥ 7.0 kPa. Subsequent inclusion of these variables in a multivariate analysis (Table 4) demonstrated that the *SREBP1c* (TT) variant and age are independent determinants of increased liver stiffness. Of note, restricting the analysis to individuals with viral hepatitis also yielded a significant (*p* = 0.014) association between the (TT) variant and TE levels >7.0 kPa (OR = 2.12, 95% confidence interval (CI) 1.16–3.87). On the other hand, we did not detect any association between this variant and liver stiffness in patients with non-viral chronic liver diseases (*p* > 0.05).

### 2.5. Sensitivity Analysis: *SREBP1c* and *PNPLA3* Variants Are Associated with Distinct Stages of Liver Injury

To further characterize the specific TE cut-offs at which the *SREBP1c* variant is associated with fibrosis, we performed a combined sensitivity analysis [5,43] of the *PNPLA3* polymorphism from our previous publication [5] and of the *SREBP1c* variant from the current study.

**Figure 3:**
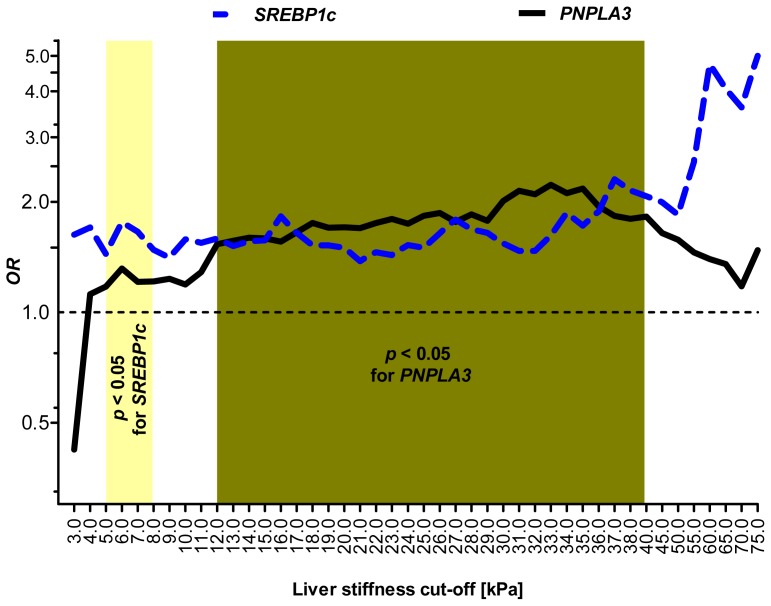
Graphical presentation of odds ratios (OR) for developing increased liver stiffness separately for the *SREBP1c* (**dotted line**) and *PNPLA3* (**black line**) variants. The OR values (log-transformed) are plotted against liver stiffness cut-off values between 3.0 and 75.0 kPa. The *SREBP1c* (T) allele represents a fibrosis risk factor at TE cut-offs between 5.0 and 8.0 kPa. On the other hand, an association between the *PNPLA3* risk allele [5] and liver fibrosis is present for more enhanced TE values (*i.e.*, between 12.0 and 40.0 kPa).

Figure 3 illustrates that the *SREBP1c* (TT) variant was associated with mild liver fibrosis, whereas the *PNPLA3* risk variant affected more advanced stages of hepatic fibrosis.

### 2.6. Combined Analysis of the Common *PNPLA3* and *SREBP1c* Variants

Finally, to assess the combined effects of the *PNPLA3* and *SREBP1c* risk variants on liver stiffness, we compared the median TE levels in carriers of none, one and both risk variants.

**Figure 4:**
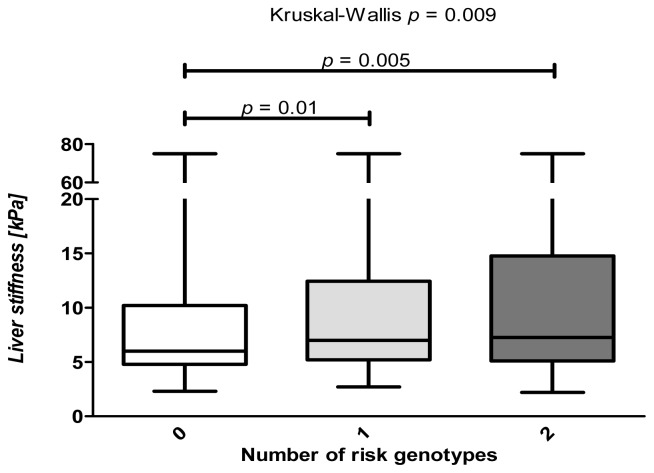
Combined analysis of the profibrogenic risk variants within the SREBP1c-PNPLA3 pathway. Liver stiffness differs significantly (*p* = 0.009, non-parametric ANOVA) between carriers with different numbers of risk genotypes (*i.e.*, *SREBP1c* (TT), *PNPLA3* (IM) or (MM)). Individuals carrying the *SREBP1c* rs11868035 and *PNPLA3* p.I148M risk variants demonstrate significantly increased TE values, as compared to individuals who do not carry any of these variants.

Figure 4 demonstrates that carriers of either *SREBP1c* or *PNPLA3*, as well as carriers of both *SREBP1c* and *PNPLA3* variants have significantly (*p* = 0.01 and *p* = 0.005, respectively) higher TE levels, as compared to individuals who do not carry any risk allele.

## 3. Discussion

Variable progression of liver fibrosis in individuals with comparable environmental risk profiles underscores the importance of genetic susceptibility in hepatic fibrogenesis [24]. To date, the p.I148M adiponutrin variant represents the only well-established proinflammatory and profibrogenic polymorphism in patients with chronic liver diseases. Here, in contrast to previous studies that investigated solely the role of the adiponutrin risk variant in liver fibrosis, we focused on the key regulator of hepatic *PNPLA3* expression. Using a candidate-gene approach, we showed that the common intronic *SREBP1c* variant, rs11868035, localized between exons 18c and 19c, is associated with liver stiffness, especially in patients presenting with low TE values. Moreover, by introducing pathway analysis, we were able to investigate a combined effect of two *SREBP1c*-*PNPLA3* polymorphisms, underscoring the effects of this specific pathway on liver stiffness.

Genetic analysis of several variants within the pathway regulating hepatic adiponutrin expression represents the major novelty of our study. This design follows the hypothesis stating that if a gene is involved in a specific process or disease process, its direct interactors might also be suspected to play a role in the same process (“disease module”) [44,45]. Pathway analysis is a novel approach, which is complementary to separate analyses of single genes, and tests if a given set of candidate genes within a metabolic pathway affects the occurrence of the disease. Given these assumptions, in the current analysis, we combined the genotyping results of the *PNPLA3* variant from our previous study [5] with the frequencies of two variants of, which is known to regulate the expression of *PNPLA3*. Based on the key regulatory function of SREBP1c [31,32], we chose two candidate variants within this locus and tested them as possible determinants of liver stiffness. Interestingly, the rs11868035 *SREBP1c* polymorphism does not influence the structure of the protein, due to its intronic localization, but Liu *et al.* [38] reported that it might affect mRNA turnover. Here, further functional studies should provide additional insights into the role of this variant in the progression of chronic liver diseases. On the other hand, others showed an involvement of SREBP1c in liver diseases, most of all, hepatic steatosis [46–48], and pointed out that activation of SREBP might be critical for the development of fatty liver [49]. Hence, one can hypothesize that carriers of the *SREBP1c* risk variant could be prone to increased fat accumulation, due to a dysfunction of the protein.

Our analysis was performed in a cohort of patients phenotyped by TE. This approach was validated in our previous study, which demonstrated that the *PNPLA3* variant is associated with increased liver stiffness [5]. The application of TE in genetic studies provides the unique opportunity to analyze the relationship between genetic variation and a wide range of liver fibrosis phenotypes (*i.e*., TE levels from 2.0 to 75.0 kPa). In the current study, this approach allowed us to identify a profibrogenic polymorphism that might be associated with early stages of liver fibrogenesis. The uni- and multi-variate analyses, including other potentially profibrogenic factors, further underscored this variant as an independent determinant of liver stiffness. Conversely to the *SREBP1c* variant, the *PNPLA3* p.I148M SNP is associated with more advanced stages of liver fibrosis and increases the risk of cirrhosis [5,13–15,17]. Especially in the setting of alcoholic liver disease or chronic hepatitis C virus infection, carriers of the p.148M variant are at risk of developing fibrosis, cirrhosis and HCC [13–23]. Of note, the TE ranges associated with the *SREBP1c* and *PNPLA3* variants do not overlap (see Tables 3 and 4 and Figure 3). Since the *PNPLA3* SNP has been previously associated with the development of HCC [18–22], further studies in patients with HCC are needed to assess whether an association between the *SREBP1c* variant and cancer susceptibility exists.

After stratification of our cohort, the association between the *SREBP1c* variant and liver stiffness was replicated in the sub-cohort of patients with viral hepatitis, but not in patients with non-viral liver disease. This observation could be related to the relatively low number of patients in the latter group and/or a lower number of individuals with non-viral diseases in the cirrhotic group; the finding could also indicate that distinct mechanisms are involved in fibrogenesis in the setting of viral and non-viral liver diseases. We did not detect a significant association between the *SREBP1c* polymorphism and histopathological fibrosis stages in the 229 individuals scheduled for this procedure, but a trend (*p* = 0.052) for different genotype distributions between F0 and F1. This lack of association might be due to the lower number of patients with histopathological assessment; furthermore, most of these patients suffered from advanced liver fibrosis [5], whereas the *SREBP1c* variant seems to be associated with lower TE results. Due to the cross-sectional design of this study, only a single TE result is available for each patient. Hence, additional studies are warranted to prospectively assess the *SREBP1c* variant and the association during disease progression. Moreover, non-invasive methods quantifying hepatic fat contents might provide additional functional insights.

## 4. Experimental Section

### 4.1. Study Cohort

The general description of the study cohort is presented in Table 5 and in our previous publication [5]. In short, the cohort consisted of 899 European individuals with chronic liver diseases (352 females, age range 17–83 years). Among included individuals, a total of 608 suffered from viral and 291 from non-viral chronic liver diseases. In these patients we assessed liver stiffness by transient elastography (TE, Fibroscan^®^, Echosens SA, Paris, France) and obtained venous blood samples after overnight fasting for routine biochemical analyses and DNA genotyping.

A subgroup of 229 patients (25.5%) was scheduled for liver biopsy, as well. This was performed according to the percutaneous Menghini technique under ultrasound guidance. Liver fibrosis in the obtained specimens was staged according to the Desmet and Scheuer score [50] by two pathologists who were blinded to the TE results. The local Ethical Committee approved the study, and all patients signed informed consent. All reported investigations were carried out in accordance with the principles of the Declaration of Helsinki, as revised in 2000.

### 4.2. DNA Isolation and Genotyping of the SREBP1c Variants

Genomic DNA was isolated from EDTA anticoagulated blood, as described previously [5]. The *SREBP1c* variants, rs2297508 (localized in exon 18c, C_3085829_20) and rs11868035 (localized between exons 18c and 19c, C_31463202_10), were genotyped using PCR-based assays with 5′-nuclease and fluorescence detection. The *PNPLA3* genotype and allele frequencies are presented in our previous manuscript [5].

### 4.3. Statistical Analysis

All tests were performed with SPSS 19.0 (IBM, Somers, NY, USA) or GraphPad Prism 5.0 (GraphPad Software, San Diego, CA, USA). The consistency of allele and genotype frequencies with Hardy-Weinberg equilibrium (HWE) was tested with exact tests (http://ihg.gsf.de/cgi-bin/hw/hwa1.pl) [27]. The association between *SREPB1c* variants and TE was analyzed in contingency tables. Allele frequencies were assessed by chi^2^ tests and genotype differences by Armitage’s trend tests [27]. Median liver stiffness values among carriers of the specific genotypes were compared by Kruskal-Wallis non-parametric analysis of variance (ANOVA) and Mann-Whitney *U* tests, as appropriate. The relationship between liver stiffness and *PNPLA3* and *SREBP1c* variants, as well as other potentially profibrogenic factors was assessed by univariate logistic regression analysis. Variables that were significant in the univariate analysis were included in the subsequent multivariate forward stepwise analysis. Finally, we performed a sensitivity analysis of the *SREBP1c* and *PNPLA3* variants, as described in our previous manuscript [5]. A two-sided *p*-value <0.05 was considered significant.

## 5. Conclusions

In summary, our study shows that variant *SREBP1c* is associated with increased liver stiffness. This observation points to a possible genetic screening strategy, comprising the *PNPLA3* and *SREBP1c* variants, to identify patients with chronic liver diseases who carry a high risk for the progression of liver disease. Moreover, it points to the genetic analysis of core fibrogenic pathways as a novel tool of risk stratification in liver fibrosis.

## Figures and Tables

**Table 1:** *SREBP1c* rs11868038 allele distribution.

Allele counts			

Transient elastography	(T)	(C)	*p*
<7 kPa	622 (66.1%)	320 (33.9%)	
≥7 kPa	604 (71.7%)	238 (28.3%)	0.009

**Table 2:** *SREBP1c* rs11868038 genotype distribution.

Genotype counts				

Transient elastography	(TT)	(CT)	(CC)	*p*
<7 kPa	204 (43.3%)	214 (45.4%)	53 (11.3%)	
≥7 kPa	217 (51.5%)	170 (40.4%)	34 (8.1%)	0.009

**Table 3:** Univariate logistic regression analyses of risk factors for developing liver stiffness ≥7.0 kPa.

Univariate analysis			

Factor	OR	95% CI	*p*
*SREBP1c* (TT)	1.658	1.035–2.656	0.035
Age	1.031	1.020–1.042	<0.001
Alcohol consumption	1.001	0.998–1.004	>0.05
*PNPLA3* (IM) + (MM)	1.179	0.955–1.456	>0.05
BMI	1.008	0.995–1.020	>0.05
Gender	0.971	0.933–1.010	>0.05

Abbreviations: BMI, body mass index; CI, confidence interval; I, isoleucine; M, methionine; OR, odds ratio; *PNPLA3*, adiponutrin; *SREBP1c*, sterol regulatory binding protein 1c.

**Table 4:** Multivariate logistic regression analyses of risk factors for developing liver stiffness ≥7.0 kPa.

Multivariate analysis			

Factor	OR	95% CI	*p*
*SREBP1c* (TT)	1.670	1.033–2.700	0.036
Age	1.031	1.020–1.042	<0.001

Abbreviations: CI, confidence interval; OR, odds ratio; *SREBP1c*, sterol regulatory binding protein 1c.

**Table 5:** Demographic and clinical data.

Variables	Subject characteristics
*N* (male/female)	899 (547/352)
BMI (kg/m^2^)	24.6 (14.9–45.2)
Age (years)	50 (17–83)

Liver disease (*N*)	
HCV	541 (60.2%)
ALD	112 (12.5%)
HBV	67 (7.5%)
PBC/PSC/AIH	67 (7.5%)
NAFLD/NASH	64 (7.1%)
Hemochromatosis	25 (2.8%)
Other liver diseases #	23 (2.6%)

Transient elastography	
Liver stiffness (kPa)	6.8 (2.2–75.0)

Values are given as medians (ranges), unless stated otherwise;

# other liver diseases include cryptogenic liver disease (*n =* 13), biliary atresia (*n* = 3), Wilson Disease (*n* = 2), amyloidosis (*n* = 1), α_1_-antitrypsin deficiency (*n* = 1), Budd-Chiari syndrome (*n* = 1), congenital liver disease (*n* = 1) and sarcoidosis (*n* = 1).

Abbreviations: AHI, autoimmune hepatitis, ALD, alcoholic liver disease; BMI, body mass index; HBV, chronic hepatitis B virus infection; HCV, chronic hepatitis C virus infection NAFLD, non-alcoholic fatty liver disease; NASH, non-alcoholic steatohepatitis; PBC, primary biliary cirrhosis; PSC, primary sclerosing cholangitis.
